# Patient experiences and health system responsiveness among older adults in South Africa

**DOI:** 10.3402/gha.v5i0.18545

**Published:** 2012-11-27

**Authors:** Karl Peltzer, Nancy Phaswana-Mafuya

**Affiliations:** 1 HIV/AIDS/STI and TB Research Programme, Human Sciences Research Council, Pretoria, South Africa; 2 Department of Psychology, University of Limpopo, Sovenga, South Africa; 3 Office of the Deputy Vice Chancellor: Research & Engagement, Nelson Mandela Metro University, Port Elizabeth, South Africa

**Keywords:** healthcare responsiveness, older adults, population survey, SAGE, South Africa

## Abstract

**Background:**

As populations age, health systems must adapt and develop approaches that meet the needs of older patients with increasing multiple chronic conditions. Understanding older populations’ perceptions of quality of care is critical to developing measures to increase the utilization of primary healthcare services. Using the data from the Global Study on Ageing and Adult Health (SAGE) survey, the current study aims to evaluate the degree of perceived responsiveness with outpatient and inpatient healthcare in South Africa.

**Methods:**

We conducted a national population-based cross-sectional study with a sample of 3,840 individuals aged 50 years or older in South Africa in 2008. The questionnaire included sociodemographic characteristics, healthcare utilization and responsiveness, and other health variables.

**Results:**

Healthcare utilization was 9% inpatient care in the past 3 years and 50% outpatient care in the past 12 months. The overall mean perceived responsiveness score for inpatient care was 71 and for outpatient care 69. According to the evaluation of inpatient care, autonomy and prompt attention showed the lowest while quality, confidentiality, and dignity showed the highest degree of perceived responsiveness among all the areas analyzed. Regarding outpatient care, prompt attention showed the lowest while quality, confidentiality, and dignity the highest degree of perceived responsiveness scores. Overall, perceived healthcare responsiveness was higher in private than in public inpatient and outpatient healthcare facilities. Multivariate analysis found that being from the White population group (OR=3.96, CI=1.54–19.19), not a public health facility (OR=0.34, CI=0.17–0.69), poor subjective health status (OR=0.53, CI=0.38–0.75) and having health insurance paying for the outpatient care visit (OR=3.39, CI=1.24–9.27) were associated with outpatient perceived healthcare responsiveness, whereas male gender (OR=0.36, CI=0.14–0.89), 80 years or older (OR=5.83, CI=1.11–30.63), being from the Indian or Asian population group (OR=8.97, CI=1.14–70.35) and not residing in a rural area (OR=0.28, CI=0.10–0.80) were associated with inpatient perceived healthcare responsiveness.

**Conclusion:**

Prompt attention, autonomy, communication and access were identified as priority areas for actions to improve responsiveness of healthcare services in South Africa.

The phenomenon of population aging has become more significant in South African society during recent decades, with the cohort aged 50 years or older increasing noticeably in both percentage and number. The social, economic and political consequences of population aging have thus become a significant factor to be taken into account with all planning aspects of policies and programmes. This is particularly the case with regard to the care of older people, in the light of the growing human immunodeficiency virus/acquired immunodeficiency syndrome (HIV/AIDS) and noncommunicable chronic disease epidemics, with the consequent additional social and economic pressures and responsibilities that have been placed on older people.

The majority of South Africans depend on the public health sector for their healthcare needs (1). In a nationally representative survey, Shisana et al. (2) found that the majority of South Africans (70%) indicated that they usually attended public healthcare (PHC) services, while 23% attended private healthcare services, and a small proportion (0.1%) utilized traditional health practitioners. In many areas of South Africa, PHC facilities are the only available or easily accessible health service facilities for local communities (3). The South African Department of Health's strategic framework for 2002–2004 identifies improvements in quality of care as one of the four key challenges currently facing the public health sector in South Africa (4).

Quality of care assessed from a patient's perspective can be measured in the form of healthcare responsiveness, which relates to patients’ experiences with the health system, with a focus on the interpersonal aspects of care. This differs from patient satisfaction which is a construct that reflects people's expectations in addition to their experiences (5).

The South African Department of Health (6) found that there was an increase in the percentage of adults who expressed dissatisfaction with all types of health services, compared to the Demographic and Health Survey (DHS) of 1998 and 2003. The level of dissatisfaction was highest in older adults (55–64 years and 65 years and older), for government hospital/clinic 22 and 29%, for private doctor 12 and 13%, and for private hospital/clinic 19 and 8%; dissatisfaction levels seemed to have been higher for public than private health services. The major reasons for dissatisfaction with public health services included long waiting times, staff attitudes, non-availability of prescribed medication and staff shortages (doctors/pharmacists), and in private health services it was long waiting times, staff attitude, and cost (6).

Previous comparable studies on healthcare responsiveness found that among older adults (50 years and above) in China, the highest ranking among seven responsiveness domains was dignity/respect, followed by quality, prompt attention, communication, confidentiality, choice, and last autonomy (7). Studies investigating factors associated with patient satisfaction in older adults included older age in USA, South Korea, Vietnam, and Greece) (8–13), higher income in USA (14), higher education in USA (8), higher levels of religious salience in USA (8), better subjective health status in USA, Vietnam, and Greece (8, 10, 12, 13), low morbidity in USA (14) and physician technical skills in USA (14).

As populations age, health systems must adapt and develop approaches that meet the needs of older patients with increasing multiple chronic conditions (15). Patients’ views are being given more and more importance in policy-making. Understanding older populations’ perceptions of quality of care is critical in developing measures to increase the utilization of healthcare services, the quality of care and the overall performance of the healthcare services. Against this background, the Study on Ageing and Adult Health (SAGE) was conducted. The aim of SAGE was to improve understanding of the health and well-being of adults aged 50 years or older in low- and middle-income countries. The countries studied were China, Ghana, India, Mexico, the Russian Federation, and South Africa. The objective of SAGE was to generate data on aging and on the health and well-being of older adults that is valid and that can be compared across countries. The study provides information on a wide range of population health, wealth, and related indicators. These indicators include household and family support networks and transfers, assets and household income and expenditure, sociodemographic characteristics, work history and benefits, health-state descriptions, anthropometrics, performance tests and biomarkers, risk factors, chronic conditions and health services coverage, healthcare use, social cohesion, subjective well-being and quality of life, and the impact of caregiving on individuals. Using the data from SA, the current study aims to evaluate the degree of responsiveness with outpatient and inpatient healthcare experiences, and to compare the experiences of individuals who used public and private healthcare services in South Africa. The resulting evidence on the latter will be used to inform policy and planning in the country.

## Methods

### Sample and procedure

We conducted a national population-based cross-sectional study with a sample of 3,840 individuals, aged 50 years or older in South Africa in 2008. The SAGE sample design entails a two-stage probability sample that yields national and subnational estimates to an acceptable precision at provincial level, by locality type (urban and rural), and by population group (including African Black, Colored, Indian or Asian, and White). SAGE was carried out in South Africa in partnership between the World Health Organization (WHO), the National Department of Health (NDOH), and the Human Sciences Research Council (HSRC). The HSRC Ethics Committee and the NDOH approved the study. Trained fieldworkers conducted face-to-face interviews with respondents.

The study sampling frame included two samples: the total of the SAGE survey to calculate health services utilization and a subsample to calculate healthcare responsiveness, that is, 1) all individuals who had been hospitalized in the previous 3 years (stayed overnight in a hospital or other type of long-term care facility) and 2) all individuals who had used an outpatient health service in the past 12 months. Only the participants who had used an inpatient and/or outpatient health service facility were requested to complete the responsiveness questions.

### Measures

The questionnaire included in this analysis used the health system responsiveness module, as developed by the WHO (5) (see Annex). Health system responsiveness scores quantify the way health systems treat people with regard to core domains. These domains, which characterize people's interactions with health systems, included the following: autonomy (involvement in decision making about personal healthcare); access (choice of healthcare provider); communication (clear explanations); confidentiality (of information); dignity (talked respectfully); prompt attention (waiting time); and quality of basic amenities (cleanliness). For each of these seven domains, one question was asked. For outpatient care and care at home ‘prompt attention (waiting time)’ was assessed with the question: ‘For your last visit to a health care provider, how would you rate the amount of time you waited before being attended to?’ For inpatient hospital ‘care quality of basic amenities (cleanliness)’ was assessed with the question: ‘For your last visit to a hospital or long-term care facility, how would you rate the cleanliness in the health facility?’ For each of the items, the degree of responsiveness was estimated from 1=very good to 5=very bad.

#### Economic or wealth status

To estimate economic or wealth status, a random effects probit model was used to identify indicator-specific thresholds that represent the point on the wealth scale above which a household is more likely to own a particular asset than not. This enabled an estimation of an asset ladder. These estimates of thresholds, combined with actual assets observed to be owned for any given household, were used to produce an estimate of household-level wealth status. This was then used to create wealth quintiles (16).

#### Overall self-rated health status

This is based on respondents’ assessment of their current health status on a 5-point scale in response to the question: ‘In general, how would you rate your health today?’ Response categories were very good, good, moderate, bad, and very bad.

#### Activity limitation

Defined as the difficulty an individual may have in executing task or actions, this was assessed with one item ‘Overall in the last 30 days, how much difficulty did you have with work or household activities?’ Response options ranged from 1=none to 5=extreme/cannot do.

#### Religious activity

Religious activities included frequency of attendance at religious services (measured on a six-point scale, from more than once a week to never). Responses were categorized into once a week or more=1 and once or twice a month to never=0.

### Data analysis

The data were entered using CSPro and analyzed using STATA Version 10. 0 (Stata Corporation, College Station, Texas, USA). The data were weighted using post-stratified individual probability weights based on the selection probability at each stage of selection. To adjust for the population distribution as represented by the UN Statistical Division and for non-response, post-stratification corrections were made to the sampling weights. Individual weights were post-stratified by province, sex, and age groups according to the 2009 Medium Mid Year population estimates from Statistics South Africa. Available at: http://www.statssa.gov.za/publications/P0302/P03022009.pdf

The first stage included a descriptive analysis of the degree of responsiveness based on a set of variables that expressed the user's degree of experience, according to five response levels (1=very good to 5=very bad). Answers of ‘refuse’, ‘don't know’, or ‘not applicable’ were recoded to missing (<1%). For each of the items, the degree of responsiveness was estimated by the mean percentage (range 0–100). For this, the scores from 0=very good to 4=very bad were reverse coded and multiplied by 25 in order to get a scale from 0 to 100, with 0 indicating the lowest and 100 the highest responsiveness. Percentages of patients’ experiences were analyzed by ‘type of care’ (outpatient or inpatient) and by public and private healthcare service utilization (17). Adding up all of the items created a responsiveness index. Since the total responsiveness index had a skewed distribution, the index was dichotomized by taking the median. Multivariate logistic regression identified (there is an omission here) based on literature review, sociodemographic (gender, age, education, wealth, population group, geolocality and religious activity) and health variables (type of health service, transportation time to the health facility, subjective health status and activity limitation) as possible predictors of healthcare responsiveness. Associations were considered significant at *P*< 0.05.

## Results

### Survey response rate

The individual response rate in this South African sample among those aged 50 years or older was 77%.

### Healthcare utilization

Of the 3,840 interviewees, 356 (9%) reported inpatient care in the 3 years prior to the survey. Of these, 70% attended a government public facility and 31% attended a privately operated health facility. Among the participants, 1,919 (50%) had received outpatient care at least once in the year prior to the interview. Of these, 73% attended a government public facility and 27% a privately operated health facility. The most common outpatient care provider (at the last outpatient visit) was a nurse (51%), followed by a medical doctor (45%), pharmacist (1%), dentist (0.6%), and others (physiotherapist, traditional health practitioner) (0.2%). Most outpatients last visited their healthcare provider last because of a chronic (on-going) condition (69%), a new condition (26%), both a chronic and new condition (4%) and for a routine check-up (1%). The most common conditions for inpatient care included a communicable disease, surgery, high blood pressure and a stroke. In the second and a third hospital visit, occupation or work related conditions were most commonly reported. In terms of outpatient care and care at home, the single most common condition was high blood pressure, which increased at repeat outpatient visits, and to a lesser extent pain in joints, diabetes and acute conditions (see Table 1).

**Table 1:** Inpatient care in the past 3 years and outpatient care and care at home in the past 12 months (%)

	Inpatient care			Outpatient care and care at home		
		
Reason for visit	Last overnight stay (*n*=356)	2nd overnight stay (*n*=86)	3rd overnight stay (*n*=49)	Last visit (*n*=1,919)	2nd visit (*n*=1,471)	3rd visit (*n*=1,228)
Communicable	13.1	9.2	4.3	2.8	2.0	1.9
Maternal	1.7	0	0	0.4	0.6	0.6
Nutrition	1.0	0	0	0.9	0.1	0.2
Acute condition	2.9	0.7	0	9.6	4.9	3.0
Injury	1.3	0	0	1.4	1.0	0.4
Surgery	13.1	4.3	2.6	0.7	0.5	0.6
Sleep problems	0	0	0	0.2	0.1	0.1
Occupation/work related	3.9	18.0	35.8	1.3	1.4	1.8
Pain in joints/arthritis	1.8	3.4	0	13.1	15.7	16.9
Diabetes	4.8	3.5	1.5	10.3	10.3	11.6
Problems with heart	5.3	11.2	5.1	3.5	2.6	2.5
Problems with mouth	0	0	0	0.9	0.2	0.4
Problems with breathing	2.5	5.4	1.9	2.9	2.7	2.8
High blood pressure	7.4	3.0	7.2	38.8	44.0	46.6
Stroke	7.4	0.9	0	0.8	1.1	1.0
Generalized pain	0	0	0	2.6	2.4	0.8
Depression or anxiety	0.1	1.7	3.3	0.7	0.8	0.9
Cancer	1.9	5.9	5.4	0.5	0.7	0.8
Other	14.3	14.2	21.4	8.5	6.7	4.4
Do not know	10.6	18.6	11.5	8.5	2.3	2.9

### Healthcare responsiveness

Results are presented by inpatient and outpatient care as well as public and private health facilities on a range from 0 (lowest responsiveness) to 100 (highest responsiveness). The overall mean perceived responsiveness score for inpatient care was 71 and for outpatient care 69. According to the evaluation of inpatient care, autonomy and prompt attention showed the lowest degree of perceived responsiveness among all the areas analyzed. The aspects related to quality, confidentiality and dignity had the highest perceived responsiveness scores. Overall, perceived healthcare responsiveness was higher in private than in public inpatient healthcare facilities. Regarding outpatient care, prompt attention showed the lowest degree of perceived responsiveness among all the areas analyzed, and the aspects related to quality, confidentiality, and dignity had the highest perceived responsiveness scores. Overall, perceived healthcare responsiveness was higher in private than in public outpatient healthcare facilities. Further, among the nine provinces in South Africa, participants from the North-West province scored the highest for inpatient healthcare responsiveness, followed by KwaZulu-Natal, Mpumalanga and Limpopo, while the Mpumalanga province scored highest for outpatient healthcare responsiveness, followed by the Western Cape, Limpopo, and KwaZulu-Natal (see Table 2).

**Table 2:** Healthcare characteristics and mean responsiveness (%)

	Total No.	Total	Public	Private	Eastern Cape	Free State	Gauteng	KwaZulu-Natal	Limpopo	Mpumalanga	North–West	Northern Cape	Western Cape
Inpatient care#			69.5	30.5									
Traveling time (1 hour or more)	258	32.9	40.0	16.8	25	56	38	19	25	23	66	63	26
Payment													
-Free	254	52.0	75.5	3.4	58	67	33	46	55	18	55	75	79
-Insurance	255	26.9	4.8	75.9	15	22	53	31	18	47	8	6	8
-Out of pocket	255	16.4	16.8	17.0	16	1	7	12	27	34	37	19	14
Responsiveness													
Prompt attention	220	68.6	65.0	77.1	61	68	62	77	76	71	78	70	69
Dignity/respect	222	73.5	70.8	79.5	70	68	70	79	75	79	80	68	73
Communication	221	69.5	67.6	73.7	65	66	63	77	73	73	80	67	70
Autonomy	219	65.4	63.6	68.9	65	61	63	74	68	68	52	65	67
Confidentiality	222	74.2	70.6	82.3	68	66	77	78	67	82	84	66	71
Access	222	71.4	68.7	77.6	65	63	72	80	77	77	76	66	68
Quality	221	75.4	77.0	71.4	66	74	70	84	81	83	91	75	72
Total responsiveness		71.4	69.4	75.8	66	67	68	78	74	76	81	68	70
Outpatient care#			73.2	26.8									
Traveling time (1 hour or more)	1,896	19.9	19.6	22.0	25	34	17	16	34	25	21	19	8
Payment													
-Free	1,890	62.7	82.1	9.6	68	52	62	54	69	58	64	87	73
-Insurance	1,893	8.4	0.3	31.6	3	9	17	3	6	9	8	3	16
-Out of pocket	1,893	26.3	15.6	58.2	25	39	13	41	25	30	29	9	11
Responsiveness													
Prompt attention	1,836	58.2	53.6	70.2	56	49	60	62	61	59	47	62	59
Dignity	1,837	70.8	67.2	80.8	63	64	70	72	75	78	72	64	73
Communication	1,830	70.5	66.9	80.4	62	66	67	72	73	78	72	63	74
Autonomy	1,828	66.4	62.7	76.6	62	65	66	67	64	72	68	60	70
Confidentiality	1,834	71.3	67.8	81.0	66	65	70	70	75	79	71	62	78
Access	1,837	69.1	65.1	80.2	65	65	65	69	70	76	70	62	75
Quality	1,837	75.1	72.1	83.9	66	67	79	74	79	79	79	67	79
Total responsiveness		68.8	65.1	79.0	63	63	68	70	71	75	69	63	73

# Cronbach alpha for the 7-item outpatients responsiveness scale was 0.92 and for the 7-item inpatient responsiveness scale 0.90 for this South African sample.

### The rank ordering of the responsiveness domains

The importance of the responsiveness domains was ranked using the overall mean results for each domain. Perceived quality of basic amenities, confidentiality and dignity/respect of treatment were the three highest ranking domains across all selected sociodemographic characteristics. The same was true for public and private outpatient care but not for private inpatient care, where perceived quality of basic amenities was rated the second lowest. For inpatient care, autonomy ranked lowest, followed by prompt attention and communication. For outpatient care, prompt attention ranked lowest, followed by autonomy and access. No gender differences were seen in ranking five domains as ‘outpatients’ and three domains as ‘inpatients’. Females ranked highest perceived quality of basic amenities while males ranked highest dignity/respect of treatment for inpatient care. There were few differences in terms of wealth status and urban or rural residents of the participants, except for rural inpatient ranking quality of basic amenities highest and urban inpatient ranking confidentiality highest. Regarding the nine different provinces, trends were similar across provinces, with a few exceptions: Eastern Cape and Western Cape ranked dignity/respect for inpatients healthcare responsiveness the highest; Gauteng, KwaZulu-Natal and Limpopo ranked access as the second highest and Northern Cape ranked prompt attention as the second highest for inpatient healthcare responsiveness. For outpatient healthcare responsiveness, Eastern Cape and Mpumalanga rated confidentiality as the highest and Free State and North-West rated communication as the second highest (see Table 3).

**Table 3:** Rankings of importance of responsiveness domain by selected sociodemographics and type of health service*

	Overall	Public	Private	Male	Female	Urban	Rural	Low wealth	High wealth	Eastern Cape	Free State	Gauteng	KwaZulu-Natal	Limpopo	Mpumalanga	North-West	Northern Cape	Western Cape
Inpatient care																		
Quality	1	1	6	3	1	3	1	1	1	3	1	4	1	1	1	1	1	2
Confidentiality	2	3	1	2	2	1	2	2	3	2	5	1	4	5	2	2	5	3
Dignity/respect	3	2	2	1	3	2	3	3	2	1	2	3	3	4	3	3	3	1
Access	4	4	3	6	4	4	4	5	5	4	6	2	2	2	4	6	6	6
Communication	5	5	5	5	5	5	5	4	4	5	4	6	5	6	5	4	4	4
Prompt attention	6	6	4	4	6	6	6	6	6	7	3	7	6	3	6	5	2	5
Autonomy	7	7	7	7	7	7	7	7	7	6	7	5	7	7	7	7	7	7
Outpatient care																		
Quality	1	1	1	1	1	1	1	1	1	2	1	1	1	1	2	2	1	1
Confidentiality	2	2	2	3	2	2	4	3	2	1	3	2	4	3	1	4	5	2
Dignity	3	3	3	2	3	3	2	2	3	4	6	3	2	2	4	3	2	5
Communication	4	4	4	4	4	4	3	4	5	5	2	4	3	4	3	2	3	4
Access	5	5	5	5	5	5	5	5	4	3	4	6	5	5	5	5	4	3
Autonomy	6	6	6	6	6	6	6	6	6	6	5	5	6	6	6	6	7	6
Prompt attention	7	7	7	7	7	7	7	7	7	7	7	7	7	7	7	7	6	7

* Where 1=highest ranking (highest importance) and 8=lowest ranking.

### Correlates of perceived healthcare responsiveness among older adults

Table 4 shows the final models from multivariable logistic regression analyses for outpatients and inpatients. Gender and age did not differ for outpatients in relation to perceived health care responsiveness. However, 80 years and older and female inpatients indicated higher perceived health care responsiveness. Wealth status and education did not play a significant role in determining older persons’ evaluation of healthcare responsiveness. Older White compared to the African Black outpatients scored significantly higher on healthcare responsiveness (OR=3.96, CI=1.54–19.19). Hospital patients residing in rural areas gave lower responsiveness ratings than hospital patients from urban areas. Outpatients and not inpatients visiting a public health facility rated their responsiveness lower than those visiting a private health facility (OR=0.34, CI=0.17–0.69). Having health insurance paid for the outpatient care visit was also significantly positively associated with healthcare responsiveness (OR=3.39, CI=1.24–9.27). For outpatients and not inpatients healthcare responsiveness significantly declined with self-rated health among the elderly people (OR=0.53, CI=0.38–0.75) (see Table 4).

**Table 4:** Multivariate logistic regression of sociodemographic and health variables on total healthcare responsiveness [Dependent Variable: Ambulatory or inpatient responsiveness]

	Outpatients		Inpatients	
		
	Mean total responsiveness		Mean total responsiveness	
		
Sociodemographics	AOR (CI 95%)	*P*	AOR (CI 95%)	*P*
Sex				
Male	1.00	0.913	1.00	0.028
Female	1.02 (0.66–1.60)		0.36 (0.14–0.89)	
Age				
50–59	1.00	0.739	1.00	0.378
60–69	1.06 (0.74–1.53)	0.373	0.56 (0.15–2.07)	0.280
70–79	1.22 (0.78–1.92)	0.736	2.11 (0.53–8.42)	0.038
80 or more	0.90 (0.48–1.69)		5.83 (1.11–30.63)	
Education				
None	1.00	0.308	1.00	0.150
Less than primary	1.36 (0.75–2.49)	0.021	3.59 (0.62–20.85)	0.341
Primary	1.64 (1.08–2.48)	0.175	2.61 (0.35–19.69)	0.582
Secondary or more	1.52 (0.82–2.80)		1.63 (0.27–9.80)	
Wealth				
Low	1.00	0.540	1.00	0.508
Medium	1.14 (0.75–1.73)	0.695	1.70 (0.34–8.56)	0.157
High	1.12 (0.62–2.05)		2.23 (0.72–6.86)	
Population group				
Black African	1.00	0.005	1.00	0.357
White	3.96 (1.54–10.19)	0.105	2.23 (0.72–6.89)	0.228
Colored	1.70 (0.89–3.24)	0.418	1.87 (0.67–5.23)	0.038
Indian or Asian	1.56 (0.52–4.66)		8.97 (1.14–70.35)	
Geolocality				
Urban	1.00	0.424	1.00	0.038
Rural	0.85 (0.57–1.27)		0.28 (0.10–0.80)	
Religious activity				
Low	1.00	0.363	1.00	0.932
High	1.22 (0.79–1.97)		1.04 (0.42–2.55)	
Health variables				
Private health facility	1.00		1.00	
Public health facility	0.34 (0.17–0.69)	0.004	0.62 (0.04–9.14)	0.721
Free healthcare	1.00		1.00	
Health insurance	3.39 (1.24–9.27)	0.018	0.35 (0.02–5.07)	0.427
Out of pocket	1.79 (0.86–3.72)	0.116	0.32 (0.09–1.19)	0.087
Time to facility				
<1 hour	1.00	0.237	1.00	0.052
≥1 hour	0.68 (0.35–1.30)		2.32 (0.99–5.40)	
Subjective health status				
Very/good	1.00	0.001	1.00	
Moderate	0.53 (0.38–0.75)	0.018	1.14 (0.24–5.36)	0.868
Bad/very bad	0.56 (0.35–0.90)		1.04 (0.16–6.81)	0.966
Activity limitation				
None	1.00	0.808	1.00	0.258
Mild	0.94 (0.57–1.54)	0.462	2.24 (0.54–9.36)	0.922
Moderate	1.18 (0.76–1.83)	0.956	0.93 (0.21–4.12)	0.787
Severe/extreme	1.02 (0.44–2.36)		1.34 (0.15–11.78)	

## Discussion

In a national sample of older adults (50 years or older) in South Africa, the study found low healthcare utilization, 9% inpatient care in the past 3 years and 50% outpatient care in the past 12 months. Myburgh et al. (18) found among outpatients 40 years and above 69% healthcare utilization in the past year in South Africa, which is higher than found in the current study among older adults.

The overall mean responsiveness score for inpatient care was 71 and for outpatient care 69, which seem to be better than in a number of other middle-income countries such as China (for inpatient: 50–54 for 60–69 and 80+ elderly and for outpatient: 53–55), India (for inpatient: 50–55 and for outpatient: 52–51) or Malaysia (for inpatient: 55–57 and for outpatient: 58–60) (7). However, it should be noted that there were some differences in the tools for assessment of responsiveness in these countries. For instance, in the Chinese study, several questions were included in each domain while only one question/domain was used in this study; factor analysis was used in the Asian studies to combine the domains while adding was used in this study to build the index. The findings on healthcare responsiveness among older adults in South Africa in this study seem to be similar to a previous study conducted in the general adult population in South Africa (68 for inpatients and 67 for outpatients) (19). However, the responsiveness was much lower than for Brazil (80 for outpatients and 76 for inpatients), and for Israel and 14 European countries (81 and higher) for both outpatient and inpatient care (17, 20, 21). The study in Brazil (17) is titled ‘healthcare satisfaction’, while in actual fact healthcare responsiveness was assessed.

The overall mean responsiveness score for outpatient and inpatient care in South Africa was lower in the public health sector compared to the private health sector. This finding may be similar to previous studies in South Africa that measured patient satisfaction (6). The finding, that having health insurance paying for outpatient care was associated with healthcare responsiveness, seems to link with the utilization of private healthcare services, which were rated higher on responsiveness.

The study found that the greatest concern of the healthcare responsiveness areas included prompt attention (waiting time), autonomy (involvement in decision making), communication (clear explanations), and access (choice of provider). In previous studies in South Africa, major reasons for dissatisfaction with the public and private health services included long waiting times (6). In a study on healthcare responsiveness among older adults in China, autonomy and access were also identified as areas of concern in healthcare responsiveness (7). Valentine et al. (5) found from general population surveys of ‘health system responsiveness’ in 41 countries that most respondents selected prompt attention as the most important domain. The finding of lack of autonomy in both inpatient and outpatient healthcare responsiveness suggests consistent failure of the South African health system to involve patients in decision-making. If greater expectation of involvement and choice and a more critical reaction to paternalistic styles are characteristic of this older adult healthcare user population, then providers will have to adapt or non-responsiveness may increase further. Understanding older populations’ perceptions of quality of care are critical in developing measures to increase the utilization of healthcare services. The results point to the need for prompt attention, autonomy, communication and access to improve responsiveness of healthcare services in South Africa.

This study found that older individuals tended to report more responsiveness with their inpatient healthcare than younger individuals, which matches previous studies (12, 13). The finding that older people reported more healthcare responsiveness may be due to differences in expectations than to any preferential treatment of the older persons. For outpatients and not for inpatients, this study found that the healthcare responsiveness significantly declined with self-rated health among the elderly people, which is in line with several other studies (8, 10, 12, 14, 22). However, activity limitation was not found to be associated with healthcare responsiveness, as also found in some studies (22). Gender and age did not differ for outpatients in relation to healthcare responsiveness, however, older and female inpatients indicated higher healthcare responsiveness. Wealth status, education and religious activity did not play a significant role in determining older persons’ evaluation of healthcare responsiveness, contrary as to what was found in some other studies (8, 14). Older White compared to African Black outpatients scored significantly higher on healthcare responsiveness, which was also found when assessing patient satisfaction among American elderly (14) and among a national sample of adults in South Africa (18).

### Study limitations

The cross-sectional study design did not permit an investigation of the cause–effect relationship between responsiveness and independent variables. Recall bias of study participants cannot be excluded, especially on the 3-year recall period for hospital admission. The study is based on a national sample meaning that respondents together have experiences from hundreds of different healthcare providers, on different levels. There is likely to be a high heterogeneity among the providers. Some score high in responsiveness and some score low. Thus, the results may not be particularly helpful for those responsible for providing care. A better design for the future could be to connect the study to catchment areas or other administrative units (districts, etc.), thus ensuring that a certain decision-maker could take care of the results and on that basis, try to improve the aspects that do not reasonably meet the populations’ desires. Ideally, such a study would have to be repeated after a period of reform, thus making it possible to examine the effectiveness in the implemented measures. Furthermore, it would have been important to also interview some staff members of health facilities and explore their experiences of working towards quality care, autonomy of the patient/person, public versus private services, etc. This could then be compared with the patient findings. It is recommended that such a study be carried out in the future.

## Conclusion

In a national sample of older adults (50 years or older) in South Africa, this study found low healthcare utilization. Overall, perceived healthcare responsiveness was higher in private than in public healthcare facilities. Prompt attention, autonomy, communication and access experiences were identified as priority areas for actions to improve responsiveness of healthcare services in South Africa.

## Annex

### Inpatient Hospital Care

**Table:** Now I want you to think again about your most recent overnight stay. I would like to ask you about your impressions of your last overnight stay. I would like you to rate your experiences using the following questions.

*For your last overnight visit to a hospital or long-term care facility, how would you rate the following:*		*Very good*	*Good*	*Moderate*	*Bad*	*Very bad*
***Q5018***	*… the amount of time you waited before being attended to?*	***1***	***2***	***3***	***4***	***5***
***Q5019***	*…your experience of being treated respectfully?*	***1***	***2***	***3***	***4***	***5***
***Q5020***	***…how clearly healthcare providers explained things to you?***	***1***	***2***	***3***	***4***	***5***
***Q5021***	***…your experience of being involved in making decisions for your treatment?***	***1***	***2***	***3***	***4***	***5***
***Q5022***	*…the way the health services ensured that you could talk privately to providers?*	***1***	***2***	***3***	***4***	***5***
***Q5023***	*…the ease with which you could see a healthcare provider you were happy with?*	***1***	***2***	***3***	***4***	***5***
***Q5024***	*…the cleanliness in the health facility?*	***1***	***2***	***3***	***4***	***5***

### Outpatient Care and Care at Home

**Table:** Now I would like you to think about your most recent visit again. I want to know your impressions of your most recent visit for healthcare. I would like you to rate your experiences using the following questions.

*For your last visit to a healthcare provider, how would you rate the following:*		*Very good*	*Good*	*Moderate*	*Bad*	*Very bad*
***Q5039***	*… the amount of time you waited before being attended to?*	***1***	***2***	***3***	***4***	***5***
***Q5040***	*…your experience of being treated respectfully?*	***1***	***2***	***3***	***4***	***5***
***Q5041***	***…how clearly healthcare providers explained things to you?***	***1***	***2***	***3***	***4***	***5***
***Q5042***	***…your experience of being involved in making decisions for your treatment?***	***1***	***2***	***3***	***4***	***5***
***Q5043***	*…the way the health services ensured that you could talk privately to providers?*	***1***	***2***	***3***	***4***	***5***
***Q5044***	*…the ease with which you could see a healthcare provider you were happy with?*	***1***	***2***	***3***	***4***	***5***
***Q5045***	*…the cleanliness in the health facility?*	***1***	***2***	***3***	***4***	***5***
